# Optimal background matching camouflage

**DOI:** 10.1098/rspb.2017.0709

**Published:** 2017-07-12

**Authors:** Constantine Michalis, Nicholas E. Scott-Samuel, David P. Gibson, Innes C. Cuthill

**Affiliations:** 1 School of Biological Sciences, University of Bristol, Bristol BS8 1TQ, UK; 2 School of Experimental Psychology, University of Bristol, Bristol BS8 1TU, UK; 3 Department of Computer Science, University of Bristol, Bristol BS8 1UB, UK

**Keywords:** defensive coloration, animal coloration, camouflage, crypsis, visual search

## Abstract

Background matching is the most familiar and widespread camouflage strategy: avoiding detection by having a similar colour and pattern to the background. Optimizing background matching is straightforward in a homogeneous environment, or when the habitat has very distinct sub-types and there is divergent selection leading to polymorphism. However, most backgrounds have continuous variation in colour and texture, so what is the best solution? Not all samples of the background are likely to be equally inconspicuous, and laboratory experiments on birds and humans support this view. Theory suggests that the most probable background sample (in the statistical sense), at the size of the prey, would, on average, be the most cryptic. We present an analysis, based on realistic assumptions about low-level vision, that estimates the distribution of background colours and visual textures, and predicts the best camouflage. We present data from a field experiment that tests and supports our predictions, using artificial moth-like targets under bird predation. Additionally, we present analogous data for humans, under tightly controlled viewing conditions, searching for targets on a computer screen. These data show that, in the absence of predator learning, the best single camouflage pattern for heterogeneous backgrounds is the most probable sample.

## 1. Introduction

Animals use a plethora of tactics to conceal themselves. The best known is background matching: an adaptation of the animal's body coloration to reduce the signal-to-noise ratio to visually hunting predators [1,2]. Through matching the hue, brightness and pattern of the background as perceived by the predator, the animal looks like and, if successful, is indistinguishable from a sample of the background [1,3–6]. One of the pioneers of the study of animal camouflage, the artist Abbott Thayer, interpreted the concept of camouflage as sampling the background quite literally, painting backgrounds through animal-shaped stencils and comparing the patterns to those of the animals [7–9]. More recent authors have grounded the definition in statistical sampling theory [1,3,4,10,11].

When the environment is homogeneous (its texture, hue and luminance do not vary) at the spatial scale of the animal, then all samples from that background are the same and there is a single optimal camouflage pattern. When the animal can occupy very different habitats or, more generally, is seen against visually distinct patch types larger than itself, then the best solution also seems clear: unless colour change is possible, match one background well at the expense of the others [12–14]. If the world consists of black and white patches, then being grey is not the answer. However, where the background is heterogeneous but the variants are more similar, a strong trade-off between matching some backgrounds at the expense of others does not necessarily hold and some form of compromise camouflage may outperform the specialist approach [6,12,13,15]. Bond & Kamil [16] investigated the effect of background heterogeneity with wild-caught birds and prey patterns able to evolve *in silico*. They concluded that when the environment consisted of large patches of two different microhabitats (course-grained, as defined by Levins [17]) then dimorphic specialists for each background evolved; but if the backgrounds varied at a spatial scale smaller than the prey (fine-grained), generalist colour morphs evolved. But what is the best generalist camouflage?

Not all samples of the background are equally effective as camouflage [6,12], even though concealment through being a random sample of the background [1] has been an influential idea in the development of a formal definition of crypsis [1,3–6]. For example, if the background has two possible variants and the animal lands on each with probability *p* and (1 − *p*), then an animal coloured to match the first variant will match its background with probability *p*, an animal with the colour of the second variant with probability 1 − *p*. An animal that is a random sample of the background will match with probability *p*^2^ + (1 − *p*)^2^ or 2*p*^2^ – 2*p* + 1. For *p* = 0.5 (and, trivially, *p* = 0 or 1), the two strategies (match one versus match randomly) are equally good, but otherwise the function 2*p*^2^ – 2*p* + 1 is concave up, so less than 1 − *p* for 0 < *p* < 0.5, and less than *p* for 0.5 < *p* < 1. Therefore, on average, matching the most common background beats (i.e. matches more backgrounds than) a random sample (also discussed by [18]).

If, as in the example above, the background has a set of states (or, more correctly, is perceived as comprising a set of states), then the modal category is the best camouflage. More generally, natural backgrounds are expected to vary continuously in colour and texture (and, for some species, polarization [19–21]), these attributes themselves having multiple perceptual dimensions. With backgrounds that vary continuously in multiple features, a prey bearing coloration equivalent to a rare background sample is more likely to mismatch its local background than a prey bearing coloration equivalent to a common sample. The concept of camouflage through being an ‘average’ sample of the background has a long history (see discussion in [6]). Thinking of the range of possible background samples as having a multivariate distribution, the best ‘average’ will be that which minimizes the deviation from all possible samples in this perceptual space. If the cost of deviation is the squared Euclidean distance, this is the familiar arithmetic mean (or centroid of the multivariate distribution). If the cost is the absolute distance, then it is the median. Of course, prey that can recognize a mismatch to their immediate background can move to a more suitable substrate (e.g. [22]), but it is still the case that a prey bearing the colours and patterns of a common background sample will have a lower cost of finding suitable resting places.

We present an analysis, based on a physiologically plausible model of low-level vision, of the colours and patterns present in a set of complex natural backgrounds, sampled at the same spatial scale as the focal prey item. All things being equal (an issue returned to in the discussion), we predict that the best camouflage patterns will be those from the centre of the sampling distribution. We tested this prediction with (i) a field experiment involving bird predation on artificial camouflaged prey items on oak trees, which have complex, highly textured, bark, and (ii) an analogous laboratory experiment with human participants searching for targets on photographs of the same bark as in the bird experiment, on a computer screen. Our experiments focus on the effects of predation rather than the cognitive and perceptual processes underlying the effects observed. We address the question of which background matching camouflage survives best under predation by multiple avian predators across a variable (but constrained) background.

## 2. Material and methods

### (a) The backgrounds

Oak tree bark was chosen as a natural complex background against which many arthropod species conceal themselves and one for which artificially camouflaged (to birds) prey can be used (e.g. [23]). Oak tree bark was photographed at approximately head height (approx. 1.75 m) and at around 1 m distance with a tripod-mounted Nikon D3200 digital SLR camera (Nikon Corp., Tokyo, Japan) in the mixed deciduous Leigh Woods National Nature Reserve, north Somerset, UK (51° 27′ 13.73″ N, 2° 38′ 2.06″ W). The 6016 × 4000 pixel digital colour images of bark included a colour standard (X-Rite Color Checker Passport; X-Rite, Grand Rapids, MI, USA) that was used for subsequent linearization and white-point balancing [24] and mapping to avian colour space [25,26]. The aperture of the camera was kept at f8 and the ISO at 100 while the shutter speed was on automatic. In total, images of the bark of 101 trees were used. Custom Matlab (The Mathworks Inc., Nattick, MA, USA) code was used both to calibrate and select an area of bark from each image. Only areas from the middle of the bark were selected, so that the tree's curvature did not distort the measurement of the texture. Each selected area had five random, but non-overlapping, samples of bark with equal dimensions (591 × 296 pixels), and these were used in subsequent analysis (totalling 505 samples of bark). Each rectangular sample was used to create a triangular artificial prey item (5 cm base and 2.5 cm height) printed at 300 dpi resolution at 1 : 1 reproduction. To select appropriate targets for each treatment, a colour and texture analysis was performed (see the electronic supplementary material).

### (b) Experimental procedure

#### (i) Field experiment

There were four treatments: (i) targets close to the cluster centres for both colour and pattern; (ii) targets far from these estimates (i.e. rare); (iii) targets that were common colour samples but rare for pattern; and (iv) targets that were rare samples for colour but common for pattern. To select the stimuli for the treatments, all the samples were ranked according to their Euclidian distances from the maximum-likelihood (ML) estimates for colour and texture, respectively. Each treatment comprised 12 different bark samples (triangular targets) from the tops or bottoms of the ranked lists as appropriate. The selections using a non-parametric distance measure [11] were identical to those using the ML estimates (see the electronic supplementary material). The experiment consisted of 10 replicate blocks, each with the same 48 target patterns (12 from each of the four treatments) placed in different areas of Leigh Woods National Nature Reserve. This, combined with the placement of our targets of each block in low densities, was done deliberately to reduce the chances that two or more blocks lay within the territory of the same individual predators. Therefore, each treatment had 12 exemplars, with each exemplar tested against 10 different trees (one in each block). Fresh targets were used for each block, but all from a single printing and calibration process.

Dead mealworms (*Tenebrio molitor* larvae frozen at −80°C, then defrosted) were pinned underneath the targets, such that only a small part of the mealworm was visible (as in [27]). Treatments were randomly placed on trees, but young trees were avoided, as were areas of bark covered with lichen. To avoid any experimenter bias in target placement, the targets were pinned at random coordinates (electronic supplementary material). Their survival was checked at 7, 24, 31, 48, 55 and 72 h. Bird predation was confirmed when all or most of the mealworm disappeared. Targets that were not predated by birds (spiders drained their fluids leaving only the exoskeleton intact, slugs and snails left slime trails, and ants cover the entire mealworm) were considered as censored data in survival analysis, along with those that could not be located or disappeared entirely, and those that survived until the end of each trial (72 h).

#### (ii) Human experiment

For this experiment, 48 bark backgrounds randomly selected from the same set used for the colour/texture analysis were used, and one random sample was extracted from each photograph. This time the samples were not dichotomized as ‘common’ or ‘rare’, but had continuous variation in how close to the densest part of the texture and colour distributions they lay. Therefore, the whole range of ‘commonness’, and not just the extremes, was tested. The size of the samples was the same as the targets from the field experiment. The target was never placed in the location it was extracted from. Consequently, 2304 (48 × 48) images were created and 48 participants were tested on 48 images each, according to a Latin square design. That is, each participant was shown a unique combination of 48 targets, balanced for order, such that all 2304 stimuli were shown once and we obtained a measure of detectability for every target on every background [28]. (The fact that there were 48 targets, as in the bird experiment, is not significant; these were a different set of samples, albeit from the same image database.)

The experiment was run as a visual search task on a linearized (gamma-corrected), 22″, 1024 × 768 pixel LaCie Electron 22Blue CRT monitor (LaCie Ltd., London) with a refresh rate of 100 Hz and a mean luminance of 72 cdm^−2^. The program was controlled using a program running Psychtoolbox 3 [28,29] for Matlab. The participants were asked to locate the target with the cursor and click on it, using the computer's touchpad (Macbook Pro; Apple Inc., Cupertino, CA, USA). This was explained with reference to an example screenshot, and then the participant gave their informed written consent in accordance with the Declaration of Helsinki. The targets had the same orientation (point up) and only one target was presented in each trial. Before all test images a black fixation cross on a mid-grey background was presented for 0.5 s. Each participant's session started with 10 practice trials and then consisted of four blocks with 12 trials in each. Trials had a time limit of 30 s, at which point the program advanced to the next image. Reaction times and accuracy (misses and time-outs) were recorded.

### (c) Analysis

#### (i) Field experiment

A survival analysis by mixed-effects Cox regression was performed on the data using the coxme function in the *coxme* package [30] in R with individual target pattern (*n* = 48) and block (*n* = 10) as random effects and, as fixed effects, the 2 × 2 factorial combination of the two-level treatments ‘colour’ (common in the background, Col+, or rare in the background, Col−) and ‘texture match’ (common in the background, Txt+, or rare in the background, Txt−). The change in deviance and degrees of freedom between the models with and without the factor in question was tested against a *χ*^2^ distribution for statistical significance [31].

#### (ii) Human experiment

Log_10_ response times were analysed with a linear mixed model (function lmer in the lme4 package, [32]), with the distances from the centroid of colour and texture distributions, respectively, as continuous fixed effects and both target and bark as random effects. The proportion of hits was analysed analogously, using a generalized linear mixed model with binomial errors (function glmer in the lme4 package). Given the Latin square design, participant is redundant as an additional random effect. The two predictors were converted to *z*-scores (i.e. centred on the mean and divided by the standard deviation) to ease comparison of effect size. Miss and timeout trials were not included in the analysis.

## 3. Results

### (a) Avian field experiment

**Figure 1.**
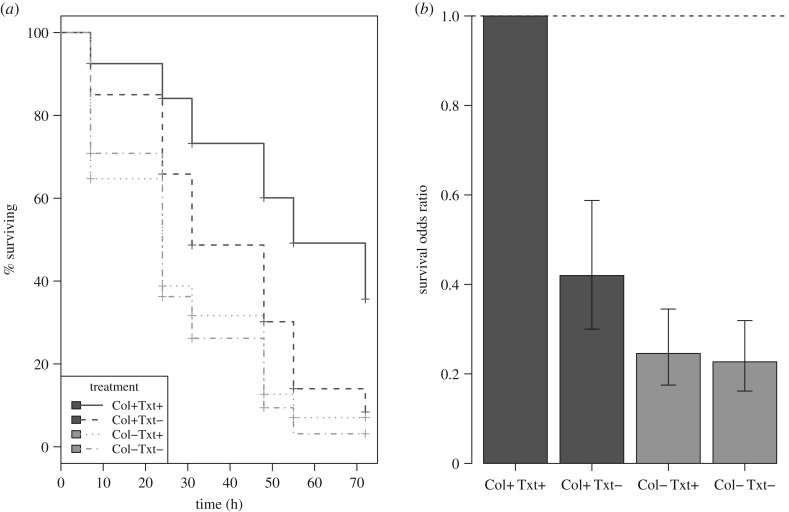
Avian field experiment. (*a*) Survival plot with respect to treatment, where targets had colours (Col) and textures (Txt) that either were common in the background (+) or rare (−). Cox regression showed that targets having common colours and texture (Col+ Txt+) survive best. (*b*) Odds of surviving relative to the best surviving treatment (Col+ Txt+, by definition plotted as 1). Values and 95% confidence intervals are from the fitted survival model.

The colour × texture interaction was significant (*χ*^2^ = 11.22, d.f. = 1, *p* = 0.0008; figure 1), and therefore the data were split by the factor ‘colour match’ and two separate analyses were performed to test the effect for texture. When the targets had background colours that were common in the environment then texture had a significant effect (*χ*^2^ = 22.72, d.f. = 1, *p* < 0.0001). When targets had background colours rare in the background, texture had no significant effect (*χ*^2^ = 0.21, d.f. = 1, *p* = 0. 6472).

### (b) Human laboratory experiment

**Figure 2.**
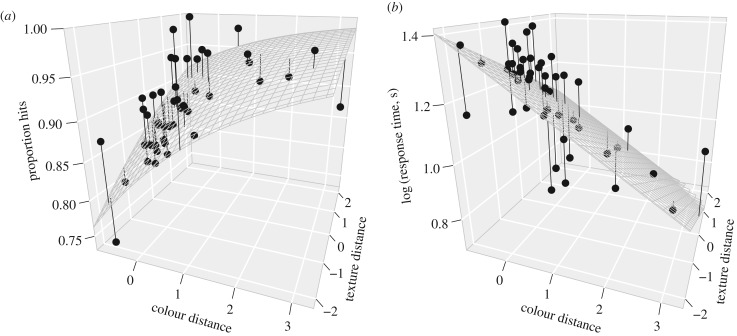
Human experiment: differences in average detectability of targets against all bark backgrounds. (*a*) Analogous data for the proportion of trials where the target was detected (hits). (*b*) Mean log_10_(response time, s) as a function of mean distance of the target from the centroid of the colour and texture distributions (both *z*-score transformed).

For response time, the colour × texture interaction was non-significant (*χ*^2^ = 1.66, d.f. = 1, *p* = 0.1980), but both colour (slope = −0.058, *χ*^2^ = 161.75, d.f. = 1, *p* < 0.0001) and texture distance (slope = −0.039, *χ*^2^ = 74.81, d.f. = 1, *p* < 0.0001) significantly decreased response times as main effects (figure 2*a*). For the proportion of hits, the interaction was also non-significant (slope = −0.039, *χ*^2^ = 1.39, d.f. = 1, *p* = 0.2391), but both colour (slope = 0.404, *χ*^2^ = 25.52, d.f. = 1, *p* = 0.0001) and texture distance (slope = 0.280, *χ*^2^ = 15.34, d.f. = 1, *p* < 0.0001) significantly increased hit rates as main effects (figure 2*b*).

## 4. Discussion

For natural backgrounds such as used in our experiments, we should not forget that the degree of background matching is not the only force at work. Because visual search in complex environments is more difficult [33], such environments are likely to be more ‘forgiving’ of imperfect camouflage, and so more morphs should be hard to find [34–39]. Nevertheless, our results indicate a large difference in the detectability of the different visual samples from the background. In both experiments, the targets that were the commonest samples from the background survived better in the face of bird predation, and were more often missed and elicited longer response times when searched for by humans, than those further from the centre of the distribution. Additionally, the field experiment showed that matching the commonest background texture and the commonest colour have non-additive effects on concealment. If the colour is rare in the background, then texture does not have an effect on the prey's survival, but if the colour is common, then bearing the commonest texture has significant advantages. However, the laboratory experiment on humans did not replicate this result: here texture and colour matching had additive effects, even though the magnitude of the effect seemed to be stronger for colour (slope seven times greater).

In the field experiment, the fact that texture matching was only important when colour also matched is plausibly explained by the observation that a difference between the colour of the prey and the background is perceived from a longer distance than any difference in texture [1,40–43]. (We use the term ‘colour’ loosely here to include achromatic differences in lightness.) However, when the predator moves closer to the prey, texture differences from the background become apparent. This can also explain why we got no such interaction in the laboratory experiment. The participants were positioned at a single distance close to the screen, and therefore texture mismatches were always, in principle, detectable. Nevertheless, the fact that the effect of texture alone did not improve a target's survival in the field experiment does not mean that matching one's environment's texture is not important. The data show that, for a target that matches the background's colour, texture matching can increase its odds of survival by approximately 45% (figure 1*b*). In principle, information on prey detection distances could be combined with different predators' contrast sensitivity functions to reveal the distance at which texture becomes important for prey detection [44,45].

One of the reasons for the choice of oak tree bark as the background was to have a set of backgrounds that are complex and varied, but all of a similar type. Those models that predict the evolution of specialist morphs rather than a single generalist [12,13] have two distinct patch types, which creates disruptive selection. Here, the aim was to investigate the situation where an animal faces a continuous distribution of possible backgrounds and evolution must pick the single colour pattern that fares best, on average. So, why do we apparently reach a different conclusion from Bond & Kamil [14], where, on fine-grained backgrounds not dissimilar to those in our experiment, multiple morphs were maintained through negative frequency-dependent selection? The answer is likely to be predator learning: in Bond & Kamil [14] prey evolution was directly coupled to predator preferences, whereas we deliberately minimized the opportunity for predator learning by placing prey at low densities and using fresh locations for each block. In the absence of the opportunity for learning, our results suggest that the single best background matching coloration is the most common sample from the population of backgrounds a prey may find itself against. However, if a population of animals have the same coloration, then predators might forage specifically for that morph and eventually the most common morph will have a reduced survival [1,46–48]. This is apostatic selection, often linked at a mechanistic level to the formation of a short-term perceptual filter, or ‘search image’, for the common prey type [49–54]. For this reason, camouflage strategies should not be considered as static optimization solutions, and the effects of learning and memory should be incorporated [55]. Predators do not only learn the distinguishing characteristics of their prey [16,55]; they are also likely to learn the statistical properties of the backgrounds on which they forage (although this has only been shown for humans [56]). Therefore, even unfamiliar prey can be detected by departures from the expected distribution of the background [56]. Whenever a predator is looking for local mismatches to the background rather than using a perceptual template for a specific conjunction of features (a search image), matching the most common background sample is likely to be favoured.

## Supplementary Material

Methods for colour and texture analysis

## Data accessibility

All data are available from Dryad [57].

## References

[1] Endler JA 1978 A predator's view of animal color patterns. Evol. Biol. 11, 319–364. (10.1007/978-1-4615-6956-5_5)

[2] Merilaita S, Scott-Samuel NE, Cuthill IC 2017 How camouflage works. Phil. Trans. R. Soc. B 372, 20160341 (10.1098/rstb.2016.0341) 28533458 PMC5444062

[3] Endler JA 1981 An overview of the relationships between mimicry and crypsis. Biol. J. Linn. Soc. 16, 25–31. (10.1111/j.1095-8312.1981.tb01840.x)

[4] Endler JA 1984 Progressive background matching in moths, and a quantitative measure of crypsis. Biol. J. Linn. Soc. 22, 187–231. (10.1111/j.1095-8312.1984.tb01677.x)

[5] Ruxton GD, Sherratt TN, Speed MP 2004 Avoiding attack. Oxford, UK: Oxford University Press.

[6] Merilaita S, Stevens M 2011 Crypsis through background matching. In Animal camouflage: mechanisms and function (eds Stevens M, Merilaita S), pp. 17–33. Cambridge, UK: Cambridge University Press.

[7] Behrens RR 1988 The theories of Abbott H. Thayer: father of camouflage. Leonardo 21, 291–296. (10.2307/1578658)

[8] Thayer GH 1909 Concealing-coloration in the animal kingdom: an exposition of the laws of disguise through color and pattern: being a summary of Abbott H. Thayer's discoveries. New York, NY: Macmillan.

[9] Thayer AH 1918 Camouflage. Sci. Mon. 7, 481–494.

[10] Endler JA 2012 A framework for analysing colour pattern geometry: adjacent colours. Biol. J. Linn. Soc. 107, 233–253. (10.1111/j.1095-8312.2012.01937.x)

[11] Endler JA, Mielke PWJ 2005 Comparing entire colour patterns as birds see them. Biol. J. Linn. Soc. 86, 405–431. (10.1111/j.1095-8312.2005.00540.x)

[12] Merilaita S, Tuomi J, Jormalainen V 1999 Optimization of cryptic coloration in heterogeneous habitats. Biol. J. Linn. Soc. 67, 151–161. (10.1111/j.1095-8312.1999.tb01858.x)

[13] Houston AI, Stevens M, Cuthill IC 2007 Animal camouflage: compromise or specialize in a 2 patch-type environment? Behav. Ecol. 18, 769–775. (10.1093/beheco/arm039)

[14] Bond AB, Kamil AC 2006 Spatial heterogeneity, predator cognition, and the evolution of color polymorphism in virtual prey. Proc. Natl Acad. Sci. USA 103, 3214–3219. (10.1073/pnas.0509963103) 16481615 PMC1413896

[15] Merilaita S, Lyytinen A, Mappes J 2001 Selection for cryptic coloration in a visually heterogeneous habitat. Proc. R. Soc. Lond. B 268, 1925–1929. (10.1098/rspb.2001.1747) PMC1088829 11564349

[16] Bond AB, Kamil AC 2002 Visual predators select for crypticity and polymorphism in virtual prey. Nature 415, 609–613. (10.1038/415609a) 11832937

[17] Levins R 1968 Evolution in changing environments. Princeton, NJ: Princeton University Press.

[18] Endler JA, Greenwood JJD 1988 Frequency-dependent predation, crypsis and aposematic coloration [and discussion]. Phil. Trans. R. Soc. B 319, 505–523. (10.1098/rstb.1988.0062) 2905489

[19] Cartron L, Josef N, Lerner A, McCusker SD, Darmaillacq A-S, Dickel L, Shashar N 2013 Polarization vision can improve object detection in turbid waters by cuttlefish. J. Exp. Mar. Biol. Ecol. 447, 80–85. (10.1016/j.jembe.2013.02.013)

[20] Sharkey CR, Partridge JC, Roberts NW 2015 Polarization sensitivity as a visual contrast enhancer in the Emperor dragonfly larva, *Anax imperator*. J. Exp. Biol. 218, 3399–3405. (10.1242/jeb.122507) 26385333

[21] Shasher N, Hanlon RT, AdM Petz 1998 Polarization vision helps detect transparent prey. Nature 393, 222–223. (10.1038/30380)9607759

[22] Kang CK, Moon JY, Lee SI, Jablonski PG 2013 Moths on tree trunks seek out more cryptic positions when their current crypticity is low. Anim. Behav. 86, 587–594. (10.1016/j.anbehav.2013.06.014)

[23] Cuthill IC, Stevens M, Sheppard J, Maddocks T, Párraga CA, Troscianko TS 2005 Disruptive coloration and background pattern matching. Nature 434, 72–74. (10.1038/nature03312) 15744301

[24] Stevens M, Parraga CA, Cuthill IC, Partridge JC, Troscianko TS 2007 Using digital photography to study animal coloration. Biol. J. Linn. Soc. 90, 211–237. (10.1111/j.1095-8312.2007.00725.x)

[25] Pike TW 2011 Using digital cameras to investigate animal colouration: estimating sensor sensitivity functions. Behav. Ecol. Sociobiol. 65, 849–858. (10.1007/s00265-010-1097-7)

[26] Stevens M, Cuthill IC 2006 Disruptive coloration, crypsis and edge detection in early visual processing. Proc. R. Soc. B 273, 2141–2147. (10.1098/rspb.2006.3556) PMC1635512 16901833

[27] Schaefer HM, Stobbe N 2006 Disruptive coloration provides camouflage independent of background matching. Proc. R. Soc. B 273, 2427–2432. (10.1098/rspb.2006.3615) PMC1634905 16959631

[28] Kleiner M, Brainard DH, Pelli GD 2007 What's new in Psychtoolbox-3? Perception 36, 14.

[29] Brainard DH 1997 The psychophysics toolbox. Spat. Vis. 10, 433–436. (10.1163/156856897X00357) 9176952

[30] Therneau TM 2015 coxme: mixed effects Cox models. R package version 2.2-5. See https://CRAN.R-project.org/package=coxme.

[31] Crawley MJ 2007 The R book. Chichester, UK: John Wiley & Son.

[32] Bates D, Maechler M, Bolker B, Walker S 2014 lme4: linear mixed-effects models using 'Eigen' and S4. R package version 1.1-7. See http://CRAN.R-project.org/package=lme4%3E.

[33] Duncan J, Humphreys GW 1989 Visual search and stimulus similarity. Psychol. Rev. 96, 433–458. (10.1037/0033-295X.96.3.433) 2756067

[34] Dimitrova M, Merilaita S 2010 Prey concealment: visual background complexity and prey contrast distribution. Behav. Ecol. 21, 176–181. (10.1093/beheco/arp174)

[35] Xiao F, Cuthill IC 2016 Background complexity and the detectability of camouflaged targets by birds and humans. Proc. R. Soc. B 283, 20161527 (10.1098/rspb.2016.1527) PMC5031667 27629039

[36] Dimitrova M, Merilaita S 2012 Prey pattern regularity and background complexity affect detectability of background-matching prey. Behav. Ecol. 23, 384–390. (10.1093/beheco/arr201)

[37] Dimitrova M, Merilaita S 2014 Hide and seek: properties of prey and background patterns affect prey detection by blue tits. Behav. Ecol. 25, 402–408. (10.1093/beheco/art130)

[38] Kjernsmo K, Merilaita S 2012 Background choice as an anti-predator strategy: the roles of background matching and visual complexity in the habitat choice of the least killifish. Proc. R. Soc. B 279, 4192–4198. (10.1098/rspb.2012.1547) PMC3441090 22915675

[39] Merilaita S 2003 Visual background complexity facilitates the evolution of camouflage. Evolution 57, 1248–1254. (10.1111/j.0014-3820.2003.tb00333.x) 12894933

[40] Barnett JB, Cuthill IC 2014 Distance-dependent defensive coloration. Curr. Biol. 24, R1157–R1158. (10.1016/j.cub.2014.11.015) 25514004

[41] Endler JA 1980 Natural selection on color patterns in *Poecilia reticulata*. Evolution 34, 76–91. (10.2307/2408316) 28563214

[42] Endler JA 1983 Natural and sexual selection on color patterns in poeciliid fishes. Environ. Biol. Fishes 9, 173–190. (10.1007/978-94-015-7682-6_7)

[43] Endler JA 1990 On the measurement and classification of colour in studies of animal colour patterns. Biol. J. Linn. Soc. 41, 315–352. (10.1111/j.1095-8312.1990.tb00839.x)

[44] Barnett JB, Scott-Samuel NE, Cuthill IC 2016 Aposematism: balancing salience and camouflage. Biol. Lett. 12, 20160335 (10.1098/rsbl.2016.0335) 27484645 PMC5014027

[45] Barnett JB et al. 2016 Stripes for warning and stripes for hiding: spatial frequency and detection distance. Behav. Ecol. 28, 373–381. (10.1093/beheco/arw168)

[46] Murdoch WW 1969 Switching in general predators: experiments on predator specificity and stability of prey populations. Ecol. Monogr. 39, 335–354. (10.2307/1942352)

[47] Oaten A, Murdoch WW 1975 Switching, functional response and stability in predator-prey systems. Am. Nat. 109, 299–318. (10.1086/282999)

[48] van Leeuwen E, Jansen VAA, Bright PW 2007 How population dynamics shape the functional response in a one-predator-two-prey system. Ecology 88, 1571–1581. (10.1890/06-1335) 17601148

[49] Tinbergen L 1960 The natural control of insects in pine woods I. Factors influencing the intensity of predation by songbirds. Arch. Neerland Zool. 13, 265–343. (10.1163/036551660X00053)

[50] Langley CM 1996 Search images: selective attention to specific features of prey. J. Exp. Psychol. 22, 152–163. (10.1037/0097-7403.22.2.152) 8618100

[51] Lawrence ES, Allen JA 1983 On the term ‘search image’. Oikos 40, 313–314. (10.2307/3544597)

[52] Pietrewicz AT, Kamil AC 1979 Search image formation in the Blue Jay (*Cyanocitta cristata*). Science 204, 1332–1333. (10.1126/science.204.4399.1332) 17813172

[53] Plaisted KC, Mackintosh NJ 1995 Visual search for cryptic stimuli in pigeons: implications for the search image and search rate hypotheses. Anim. Behav. 50(Pt5), 1219–1232. (10.1016/0003-3472(95)80039-5)

[54] Reid PJ, Shettleworth SJ 1992 Detection of cryptic prey: search image or search rate? J. Exp. Psychol. 18, 273–286. (10.1037/0097-7403.18.3.273) 1619395

[55] Troscianko J, Lown AE, Hughes AE, Stevens M 2013 Defeating crypsis: detection and learning of camouflage strategies. PLoS ONE 8, e73733 (10.1371/journal.pone.0073733) 24040046 PMC3769369

[56] Chin X, Hegdé J 2012 Learning to break camouflage by learning the background. Psychol. Sci. 23, 1395–1403. (10.5061/dryad.q4b78) 23064405

[57] Michalis C, Scott-Samuel NE, Gibson DP, Cuthill IC 2017 Data from: Optical background matching camouflage. *Dryad Digital Repository*. (10.5061/dryad.q4b78) PMC5524497 28701559

