# Supplementary material for: Optimal background matching camouflage
Source: Proc Biol Sci. 2017 Jul 12;284(1858):20170709. doi: 10.1098/rspb.2017.0709 (PMC5524497; doi:10.1098/rspb.2017.0709)
Supplement: Methods for colour and texture analysis [file rspb20170709supp1.docx]

**Supplementary Material for Michalis et al. Optimal Background Matching**

**Methods**

**(a) The backgrounds**

As described below, each sample was reduced to three numbers describing its mean colour and 24 numbers describing its achromatic texture (spatial variation in lightness).

**(i) Colour analysis**

The colour space of the blue tit (*Cynanistes caeruleus*) was modelled as this and other passerine species have been seen predating the artificial prey in previous experiments and the spectral sensitivity of its retinal cones cells are known [1]. First the mean RGB pixel values for each bark sample (n=505) were transformed to relative photon catches of the blue tit’s cones under a D65 standard daylight illuminant [2]. D65 was used because the field experiment was carried out in winter, with targets illuminated by a mixture of skylight and direct sun [3]. An avian analogue of human L*a*b* colour values, henceforth called ‘pseudo-Lab’, was then used for the analysis. The original L*a*b* colour space has dimensions representing luminance (L), red-green (a*) and yellow-blue (b*) opponent channels and can represent all human-perceived hues [4]. It is more appropriate for our purposes than calculations based on RGB pixel values since the differences or sums of cone outputs, and not their separate signals, are processed by nervous systems [4, 5]. The double cone photon catch was used as a avian proxy for human luminance [6]; the ratio of (L – M) to (L + M) photon catch as a red-green opponent channel; and the ratio of (M + L – 2*S) to (S + M + L) photon catch as a yellow-blue opponent channel. Each channel/dimension was scaled to lie between 0 and 1 (for L, black = 0 and 1 = white; for a, 0 = green, 1 = red; for b, 0 = blue, 1 = yellow). Note that the bird’s UV sensitivity was ignored, which in this case is a reasonable approximation since the oak bark and these artificial prey lacked any UV reflection [2, 7]. Although it is not known whether birds have such opponent channels, birds certainly have excellent colour discrimination along these dimensions [8-10] and, because these dimensions are orthogonal, they are a concise description of the range of perceived colours (see discussion in[11])

Finally, the distance of each sample’s colour from the most densely occupied region of colour space (i.e. the commonest colours) was estimated. This was done both parametrically and non-parametrically. For the parametric measure, the maximum likelihood estimates of the mean colour (pseudo-Lab) vector were estimated using the mlest function in the mvnmle package [12] in R 3.1.3 (The R Foundation for Statistical Computing, Vienna, Austria). This assumes multivariate normality, which one would not generally expect to hold [13]. However, from inspection of the observed distributions, although not strictly true, the departures from normality were unlikely to bias the analysis (kurtosis was higher than Gaussian but skew was only 0.8, -0.7 and 2.9 for L, a and b respectively). The Euclidean distance of each sample’s pseudo-Lab values from the maximum likelihood pseudo-Lab estimates (i.e. from the centres of the colour space) was measured (Figure S1a). The samples with the shortest distance to the maximum likelihood estimates (which were also the samples nearest the centroid) had the commonest colours. The second, non-parametric, measure was based on first measuring the distance of each point to every other point in colour space, and then calculating the median of these distances for each point. This is essentially Mielke & Berry’s [14] distance measure, as utilised by Endler & Mielke [13] to compare the similarity of distributions of colours. The correlation between the parametric and non-parametric measures was 0.986. We stress again that there is no reason to expect such strong agreement in general but, for our colours, which were averages for the target-sized samples, the parametric and non-parametric measures yield the same results.

**(b) Texture analysis**

In order to quantify the visual texture of tree barks, log-Gabor filters of different special scales and orientations were used. We chose this method over other texture descriptors [e.g. 15, 16-19] because of a combination of simplicity, computational efficiency and link to neural processing (see below). Gabor wavelets were introduced by David Gabor [20] and since then they have been used as a method for extracting features of a substrate’s texture using images [21]. A Gabor filter is created by the multiplication of a Fourier transform and a Gaussian distribution. As a result, Gabor filters have spatially localised patterns, in comparison with the Fourier transform, which extends spatially to infinity. Daugman [22] linked the way that Gabor filters process visual information with the responses of certain simple cells in the visual cortex of mammalian brains. Both can perceive the structure of a pattern by detecting changes in luminance at specified spatial scales and orientations. (The same principles can be applied to chromatic variation, but the greatest textural information in natural scenes is usually achromatic.) [23];[24];[6]. Field [25] went on to show that a Gaussian of the log-frequency (a log-Gabor filter) has better statistical properties than a simple Gabor and matches the image statistics of a natural scene more closely.

For the analysis, the pseudo-luminance planes of each bark sample were convolved with log-Gabor filters of four different spatial frequencies (a wavelength equivalent to 1, ½, ¼ and 1/8^th^ of the target’s width) and six orientations (0 to 150° in 30° increments). Therefore, the texture of each bark sample was described by twenty-four dimensions (one for each filter). We used the same approach as in our colour analysis to determine both a parametric and non-parametric measure of the distance of each point from the centre of 24D ‘texture space’. First, the Euclidian distance between all samples and the maximum likelihood estimates for each dimension of the 24D space. This again assumes multivariate normality, but skewness was low (range from -0.29 to 1.19 (median 0.01) across the 24 variables) as was kurtosis (range from -0.39 to 2.74, median 0.18). To visualize the sources of texture variation, multidimensional scaling (MDS), using function cmdscale in R, was used to reduce the twenty-four dimensional data of the texture to two dimensions. Under this transformation, the commonest textures are near the origin and the least common ones are those further away from the origin (Figure S1a). Note that the MDS was for visualisation purposes only; the distance calculations were based on the full 24D space. That said, the MDS indicates that the main variation between target-sized bark samples lay in the relative representation of high and low spatial frequencies (fine and coarse grained structure, respectively) and the relative representation of vertical and horizontal patterning (Figure S1b). The second, non-parametric, measure of the distance of each point from the centre of texture space was the median distance from each point to all other points in the 24D space. This is a textural equivalent to Endler & Mielke’s within-group distance measure for colour. The correlation between the parametric and non-parametric measures was 0.983.

The colour patterns corresponding to each treatment were selected and triangular artificial targets were printed with a Hewlett Packard Colour Laserjet 2500 (300 dots per inch) printer, calibrated such that the average printed colours matched the average colour of bark as specified by the calculated single and double cone catches from spectrometry [as in 26]. The targets were pinned on trees randomly using a 25cm x 25cm quadrat divided into 10 x 10 locations and a table with two randomly generated numbers (0-9) for each treatment of each block. The numbers were treated as coordinates and, with the help of the quadrat, these coordinates determined placement of each target.


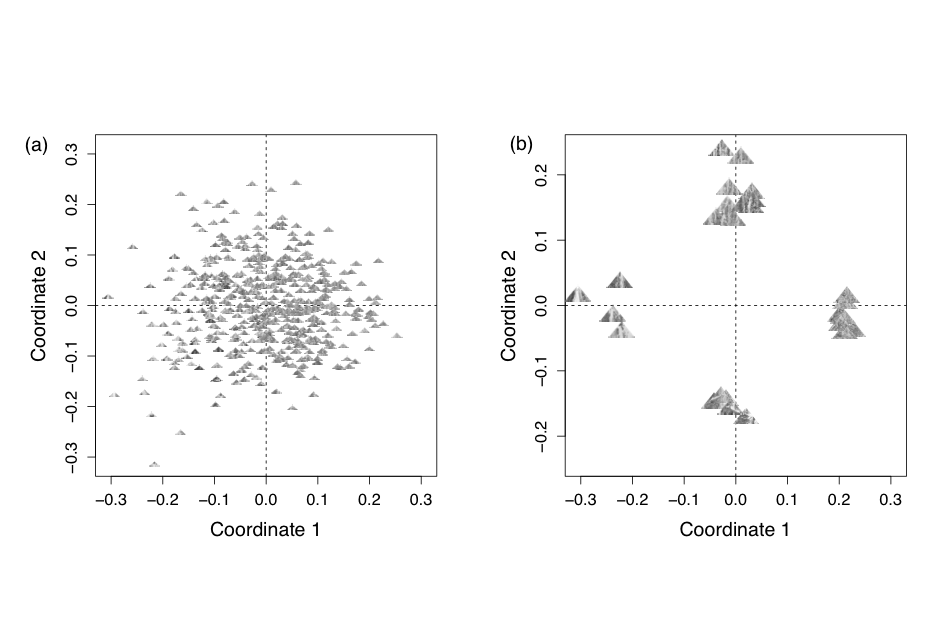


Figure S1 (a) Distribution of textures of bark samples, reduced (for visualisation) from 24 dimensions to two using Multidimensional Scaling (MDS). Coordinate 1 captures variation in dominant spatial frequency (-ve: lower spatial frequencies; +ve higher spatial frequencies); coordinate 2 captures variation in dominant orientation (-ve: horizontal textures; +ve vertical textures). (b) The extremes of the variation in the MDS dimensions, enlarged to help visualise the nature of the dimensions.

**Supplementary references**

1. Hart NS, Partridge JC, Cuthill IC, Bennett ATD. 2000 Visual pigments, oil droplets, ocular media and cone photoreceptor distribution in two species of passerine: the blue tit (*Parus caeruleus* L.) and the blackbird (*Turdus merula* L.). *J Comp Physiol A* **186**, 375-387. (doi:10.1007/s003590050437).

2. Stevens M, Cuthill IC. 2006 Disruptive coloration, crypsis and edge detection in early visual processing. *Proc R Soc B-Biol Sci* **273**(1598), 2141-2147. (doi:10.1098/rspb.2006.3556).

3. Endler JA. 1993 The color of light in forests and its implications. *Ecol Monogr* **63**(1), 1-27. (doi:10.2307/2937121).

4. Wyszecki G, Stiles WS. 1982 *Color Science: Concepts and Methods, Quantitative Data and Formulae*. 2nd ed. New York, John Wiley.

5. Kelber A, Vorobyev M, Osorio D. 2003 Animal colour vision - behavioural tests and physiological concepts. *Biol Rev* **78**(1), 81-118. (doi:10.1017/S1464793102005985).

6. Jones CD, Osorio D. 2004 Discrimination of oriented visual textures by poultry chicks. *Vision Res* **44**, 83-89. (doi:<http://dx.doi.org/10.1016/j.visres.2003.08.014>).

7. Cuthill IC, Hiby E, Lloyd E. 2006 The predation costs of symmetrical cryptic coloration. *Proc R Soc B-Biol Sci* **273**, 1267-1271.

8. Osorio D, Jones CD, Vorobyev M. 1999 Accurate memory for colour but not pattern contrast in chicks. *Curr Biol* **9**(4), 199-202. (doi:10.1016/S0960-9822(99)80089-X).

9. Kelber A, Osorio D. 2010 From spectral information to animal colour vision: experiments and concepts. *Proc R Soc B-Biol Sci* **277**(1688), 1617-1625.

10. Osorio D, Vorobyev M, Jones CD. 1999 Colour vision of domestic chicks. *J Exp Biol* **202**, 2951-2959.

11. Xiao F, Cuthill IC. 2016 Background complexity and the detectability of camouflaged targets by birds and humans. *Proc R Soc B-Biol Sci* **283**(1838), 6. (doi:10.1098/rspb.2016.1527).

12. Gross K, Bates D. 2012 *mvnmle: ML estimation for multivariate normal data with missing values. R package version 0.1-11.* [*https://CRAN.R-project.org/package=mvnmle*](https://CRAN.R-project.org/package=mvnmle).

13. Endler JA, Mielke PWJ. 2005 Comparing entire colour patterns as birds see them. *Biol J Linnean Soc* **86**, 405-431. (doi:10.1111/j.1095-8312.2005.00540.x).

14. Mielke PWJ, Berry KJ. 2007 *Permutation Methods: A Distance Function Approach.* Berlin, Springer.

15. Endler JA. 2012 A framework for analysing colour pattern geometry: adjacent colours. *Biol J Linnean Soc* **107**(2), 233-253. (doi:10.1111/j.1095-8312.2012.01937.x).

16. Stoddard MC, Kilner RM, Town C. 2014 Pattern recognition algorithm reveals how birds evolve individual egg pattern signatures. *Nat Commun* **5**, 4117. (doi:10.1038/ncomms5117).

17. Stoddard MC, Stevens M. 2010 Pattern mimicry of host eggs by the common cuckoo, as seen through a bird's eye. *Proc R Soc B-Biol Sci* **277**(1686), 1387-1393. (doi:10.1098/rspb.2009.2018).

18. Portilla J, Simoncelli EP. 2000 A parametric texture model based on joint statistics of complex wavelet coefficients. *Int J Comput Vis* **40**(1), 49-71. (doi:10.1023/a:1026553619983).

19. Westmoreland D, Kiltie RA. 1996 Egg crypsis and clutch survival in three species of blackbirds (Icteridae). *Biol J Linnean Soc* **58**(2), 159-172.

20. Gabor D. 1946 Theory of communication. *J Inst Electr Eng 3* **93**, 429–457.

21. Daugman JG, Kammen DM. 1986 Pure orientation filtering: A scale invariant image-processing tool for perception research and data compression. *Behav Res Methods Instrum Comput* **18**, 559–564. (doi:10.3758/BF03201429).

22. Daugman JG. 1985 Uncertainty relation for resolution in space, spatial frequency, and orientation optimized by two-dimensional visual cortical filters. *JOSA A* **2**(7), 1160–1169. (doi:10.1364/JOSAA.2.001160).

23. Logothetis NK, Schiller PH, Charles ER, Hurlbert AC. 1990 Perceptual deficits and the activity of the colour-opponent and broad-band pathways at isoluminance. *Science* **247**(4939), 214-217. (doi:10.1126/science.2294602).

24. Osorio D, Miklosi A, Gonda Z. 1999 Visual ecology and perception of coloration patterns by domestic chicks. *Evol Ecol* **13**(7-8), 673-689. (doi:10.1023/a:1011059715610).

25. Field DJ. 1987 Relations between the statistics of natural images and the response properties of cortical cells. *JOSA A* **4**(12), 2379-2394. (doi:10.1364/josaa.4.002379).

26. Cuthill IC, Stevens M, Sheppard J, Maddocks T, Párraga CA, Troscianko TS. 2005 Disruptive coloration and background pattern matching. *Nature* **434**, 72-74.
